# Supplementary material for: ZeOncoTest: Refining and Automating the Zebrafish Xenograft Model for Drug Discovery in Cancer
Source: Pharmaceuticals (Basel). 2019 Dec 24;13(1):1. doi: 10.3390/ph13010001 (PMC7169390; doi:10.3390/ph13010001)
Supplement: Supplementary file 1 [file pharmaceuticals-13-00001-s001.zip › SupplementaryMaterial_ProofRead/SupplementaryFigure1.pdf]

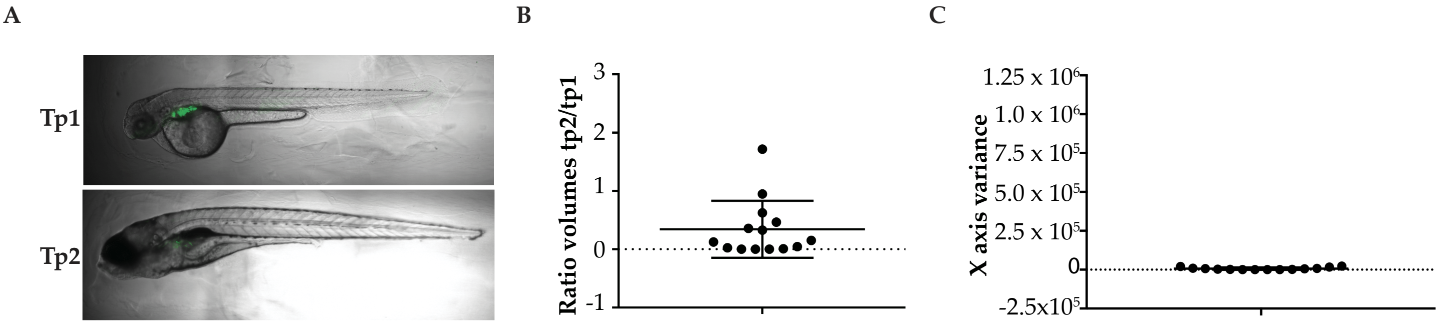

Figure S1. Evaluation of growth and dispersion in BJ non-transformed control cells xenografts. (A) Representative images of injected embryos at tp1 and tp2. (B) Scatter dot plots of the ratios of volumes between tp1 and tp2. (C) Scatter dot plot of the x variance at tp2 of injected embryos. Each dot in the graphs represents the measurement of a fish.
