# Supplementary material for: ZeOncoTest: Refining and Automating the Zebrafish Xenograft Model for Drug Discovery in Cancer
Source: Pharmaceuticals (Basel). 2019 Dec 24;13(1):1. doi: 10.3390/ph13010001 (PMC7169390; doi:10.3390/ph13010001)
Supplement: Supplementary file 1 [file pharmaceuticals-13-00001-s001.zip › SupplementaryMaterial_ProofRead/SupplementaryFigure2.pdf]

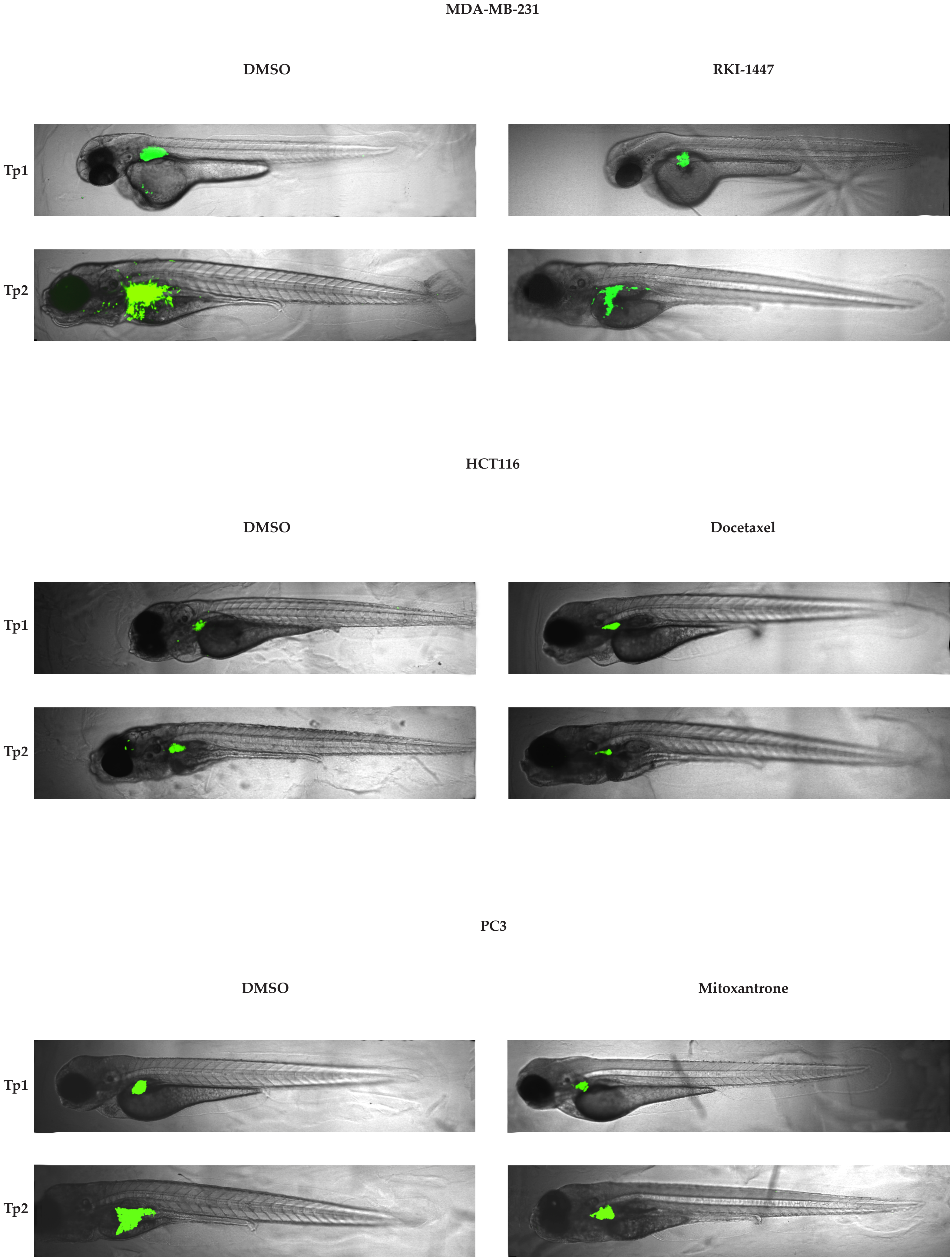

Figure S2. Representative images of fishes injected with the 3 cell lines and treated with the respective drugs.
