# Supplementary material for: ZeOncoTest: Refining and Automating the Zebrafish Xenograft Model for Drug Discovery in Cancer
Source: Pharmaceuticals (Basel). 2019 Dec 24;13(1):1. doi: 10.3390/ph13010001 (PMC7169390; doi:10.3390/ph13010001)
Supplement: Supplementary file 1 [file pharmaceuticals-13-00001-s001.zip › SupplementaryMaterial_ProofRead/SupplementaryTable1.pdf]

Nº	PMID	Stage	Tº	Injection Site	Cell Labelling	Individualization	MoA
1	15968639	3.5hpf	31	Blastodisc	AD infection	No	No
2	17051341	2dpf	35	Yolk sac	CM-DiI	No	No
3	16892036	3hpf	31	Blastodisc	AD infection	No	Yes
4	17409396	2dpf	28	PVS/DoC	WISH/Dye	No	Yes
5	17195184	4-10hpf	26	Neural plate	WISH	No	Yes
6	17875720	3.5hpf	30	Blastodist	Infected (pDsRed2- C1)	No	Yes
7	18007628	2dpf	28	PVS/DoC	WISH/Dye	No	No
8	17954920	25-35 dpf	28	Peritoneal	Stable transfection	No	Yes
9	18451167	3.5-4.5hpf	31	Yolk sac	Stable transfection	No	No
10	19199503	6hpf	28	Yolk sac	Luciferase	No	No
11	19400945	2dpf	35	Yolk sac	CM-DiI	No	No
12	19685150	2dpf	28	Yolk sac	Stable transfection or QTracker Red kit	No	Yes
13	19747919	2dpf	35	Yolk sac	CM-DiI	No	Yes
14	19887629	2dpf	28	PVS	DiI	No	Yes
15	20047470	2dpf	35	Yolk sac	CM-DiI	No	Yes
16	19627396	2dpf	28	PVS/DoC	WISH	No	yes
17	20339318	4-6 months & 5dpf	28.5	Yolk sac/PVS	zRag2-EGFP-mMyc construct	No	No
18	20530574	2dpf	35.5	Common cardinal vein	Stable expression vectors	No	Yes
19	20630586	3hpf	35	Blastodisc	Gold nanoparticles & CM-DiI	No	No
20	21127485	2dpf	28	PVS	DiI	No	No
21	24213127	24hpf	28.5	DoC	CM-DiI	No	Yes
22	21228037	2dpf	34	Posterior cardinal vein	CM-DiI	No	Yes
23	21423205	4hpf	----	Under cell mass	Fusion GFP protein & WISH	No	Yes
24	21517816	2dpf	35	Yolk sac	CM-DiI	No	No
25	21618587	2dpf	35	Yolk sac	CM-DiI	No	No
26	21622720	2dpf	----	PVS	CM-DiI	No	Yes
27	21744342	2dpf	28	Yolk sac	CM-DiI	No	Yes
28	21765912	2dpf	28	PVS	Stable transfection	No	Yes
29	21976976	2dpf	28	Yolk sac	Stable transfection	No	No
30	22033190	2dpf	34	Yolk sac	CM-DiI	No	No
31	22195560	2dpf	31	Yolk sac	DiI	No	Yes
32	21515914	2dpf	----	Pericardial	CM-DiI	No	Yes
33	22194464	2dpf	34	Yolk sac	Cell tracker Green CMFDA	No	No
34	21671725	2dpf	33	Spinal cord	Q-Tracker 605 Cell Labeling kit	No	Yes
35	22347456	2dpf	34	Yolk sac	CM-DiI	Yes	No
36	22374800	2dpf	34	DoC	CM-DiI	No	Yes
37	22569777	2dpf	31	Yolk sac	DiI	No	No
38	22711017	2dpf	28	PVS	Stable (pcDNA3.0-DsRed)	No	No
39	23158001	2dpf	32	PVS	Stable transfection	No	Yes
40	22183788	4dpf & 10dpf	29	Yolk Sac	CM-DiI	No	Yes
41	23250956	2dpf	34	Pericardial	Stable transfection	No	Yes
42	23261760	2dpf	35	Yolk sac	Stable transfection	No	Yes
43	23429286	7dpf	34	Brain peri-ventricular	Stable transfection	No	Yes
44	23581411	2dpf	28	PVS	CM-DiI	No	Yes
45	23594209	2dpf	28	PVS	Q Tracker kit	No	No
46	23613942	2dpf	35	Yolk sac	Stable transfection	No	Yes
47	23618854	2dpf	34	DoC	CM-DiI	No	Yes
48	23688428	2dpf	----	Yolk sac	CM-DiI	No	Yes

49	23689123	3dpf	37	Pericardium	Cell tracker Orange CMTMR	No	Yes
50	23807209	2dpf	33	Yolk sac	CM-DiI	No	Yes
51	23835085	2dpf	----	Intra-vitreal	Stable transfection	Yes	No
52	23874489	2dpf	----	PVS	Vybrant CiI	Yes	No
53	23899555	2dpf	28	PVS	-----	Yes	Yes
54	24165931	2dpf	----	PVS	CM-DiI	No	No
55	24196484	2dpf	33	DoC	CM-DiI & stable transfection	No	Yes
56	24290981	2dpf	----	PVS & DoC	DiI	Yes	Yes
57	23623984	2dpf	32	Cardinal vein	SNARF-1 (cell dye)	No	Yes
58	23973329	2dpf	33	DoC	Red fluorescent cell tracer	No	Yes
59	24089705	2dpf	34	PVS	CM-DiI	No	Yes
60	24056961	1dpf	35	Yolk sac	CMTMR	No	Yes
61	24154958	2dpf	33-34	Subcutaneous or DoC	Quantum dots QD605	No	Yes
62	24416389	2dpf	----	Yolk sac	CM-DiI	No	Yes
63	24454867	2dpf	32	Yolk sac	Stable transfection	No	No
64	24454929	2dpf	35	Yolk sac	Stable transfection	Yes	No
65	24461128	24-30hpf	35	Yolk sac	Cell Brite DiD	No	Yes
66	24556065	2dpf	31	Yolk sac	DiI	Yes	No
67	24830720	2dpf	28	PVS	DiI	No	Yes
68	24974828	2dpf & 35dpf	34	Yolk sac & Eye	CM-DiI	Yes	Yes
69	25066122	2dpf	34	Pericardial	CFSE	No	Yes
70	25117453	2dpf	32	Yolk sac	Stable transfection	No	Yes
71	25209178	Adult & 2dpf	34 &32	Dorsum aorta & Yolk sac	Stable transfected	No	No
72	25249605	2dpf	34	Yolk sac	Stable transfected	Yes	Yes
73	25281505	2dpf	----	Yolk sac	CM-DiI	No	Yes
74	25281719	2dpf	34	Yolk sac	Stable transfected	No	No
75	25397870	2dpf	31	Yolk sac	DiI	No	Yes
76	25477335	2dpf	28	Yolk sac	CM-DiI	No	Yes
77	25504881	1dpf	35	Yolk sac	CM-DiI	No	Yes
78	25551022	2dpf	32	Common cardinal vein	Stable expresión vectors	No	No
79	24947063	2dpf	28	PVS	---------------	No	No
80	24976296	36hpf	----	Yolk sac	Stable transfected	No	Yes
81	25388286	2dpf	34	Yolk sac	CM-DiI	No	Yes
82	25609010	2dpf	28	Brain ventricle	Cell Brite DiD	No	No
83	25609197	2dpf	33	DoC	Stable transfected	No	No
84	25624101	2dpf	34	Yolk sac	CM-DiI	No	Yes
85	25697483	2dpf	32	Cardinal vein	SNARF-1 (cell dye)	No	Yes
86	25768009	2dpf	34	Yolk sac	CM-DiI or CMFDA	No	No
87	25772246	2dpf	----	Yolk sac or DoC	QTracker cell labelling kit	No	Yes
88	25818410	2dpf	32	Yolk sac	Stable transfection	No	Yes
89	25826087	2dpf	28	PVS	DiI	No	Yes
90	25849225	2dpf	33	DoC	Red fluorescent cell tracer	No	No
91	26035715	2dpf & 6months	35.5 & 28	PVS	CM-DiI	No	Yes
92	26123890	2dpf	28	PVS	Hoechst 33342 or Cell Tracker green	No	Yes
93	26169357	2dpf	28.5	Vitreous cavity	DiI	Yes	Yes
94	26313918	2dpf	29	PVS	Stable transfection	No	Yes
95	26388134	1dpf	34	PVS	CM-DiI	No	No
96	26412466	2dpf	31	Yolk sac	CM-DiI	No	Yes
97	26449749	2dpf	----	DoC	PKH26 fluorescent cell linker	No	No
98	26476432	1dpf	35	Yolk sac	CMTMR	No	Yes
99	26498353	2dpf	35	Yolk sac	CM-DiI	No	Yes

100	26672745	2dpf	28.5	Yolk sac	Stable transfection	No	No
101	25577646	2dpf	34	Yolk sac	DiO green dye	No	No
102	25991856	2dpf	28	PVS	DiI	No	Yes
103	25519702	2dpf	28	PVS	Red-fluorescence-labeled	No	Yes
104	25858144	2dpf	33	DoC	Stable transfection	No	Yes
105	25492861	2dpf	28	PVS	DiI or Vybrant DiD	No	Yes
106	26483278	2dpf	32	PVS	DiI	No	Yes
107	26310813	2dpf	28.5	PVS	DiI	No	Yes
108	26650921	2dpf	34	Yolk sac or DoC	PKH26 fluorescent cell linker	No	No
109	26657275	3dpf	28	PVS	--	No	No
110	26659251	36hpf	32	Hindbrain-midbrain	Stable transfection	No	No
111	26741506	2dpf	34	Yolk sac	CM-DiI	No	Yes
112	26744352	2dpf	34	DoC	Stable transfection	No	Yes
113	26746804	Adult & 2dpf	34 & 32	Dorsal aorta & Yolk sac	Stable transfection	No	No
114	26762853	2dpf	----	PVs	FAST DiI	Yes	Yes
115	26829331	2dpf	28	PVs	DiI	No	No
116	27036136	2dpf	32.5	PVs	DiI	No	Yes
117	27049037	2dpf	33	Yolk sac	CM-RED	No	No
118	27091969	3dpf	----	Pericardial	CellMask Deep Red or Hoechst 33342	No	No
119	27113436	2dpf	33	DoC	Stable transfection	No	Yes
120	27158859	2-3dpf	33±1	Hindbrain ventricle	CM-DiI	No	No
121	27197202	2dpf	33	DoC	CM-DiI & stable transfection	Yes	Yes
122	27199173	2dpf	35	DoC	Stable transfection	No	Yes
123	27207793	2-4dpf	----	PVS	CM-DiI	No	No
124	27242319	0.5-2dpf	31	Yolk sac	Stable transfection	No	No
125	27247548	52hpf	31	Brain	Stable transfection or CiI or CiO	No	No
126	27258728	2dpf	32	Yolk sac	CM-DiI	No	No
127	27427902	2dpf	31	Yolk sac	CiI	No	No
128	27434411	2dpf	35	DoC	CM-DiI	No	Yes
129	27457520	2dpf	28	PVS	DiI	No	Yes
130	27466505	2dpf	35	Hindbrain ventricle	CFSE	No	Yes
131	27504667	2dpf	34	DoC	Stable transfection	No	No
132	27517156	2dpf	34	PVS	CM-DiI	No	Yes
133	27556456	2dpf	34	DoC	CFSE	No	No
134	26804176	2dpf	----	PVS	CM-DiI	No	Yes
135	27785023	2dpf	----	Yolk sac	CM-DiI	No	No
136	27825113	2dpf	35	Yolk sac	Stable transfection	No	Yes
137	27835901	2dpf	33	DoC	mCherry fluorophore	No	Yes
138	27879396	2dpf	33	Yolk sac	DiI or DiO	No	No
139	27906672	2dpf	30.5	DoC	Stable transfection	No	Yes
140	27924011	2dpf	35	Yolk sac	CM-DiI	No	Yes
141	27989824	2dpf	34	Yolk sac	DiI	No	No
142	27006469	2dpf	28.5	Yolk sac	Red fluorescence	No	Yes
143	26744527	2dpf	----	Cardiac	DiD or DiO	Yes	No
144	27464807	2dpf	34	DoC	CM-DiI	No	No
145	27481363	2dpf	32	PVS and Yolk sac	CM-DiI	No	No
146	28043811	2dpf	----	Yolk sac	CM-DiI	No	Yes
147	28075592	2dpf	35	Yolk sac	CM-DiI	No	No
148	28088004	2dpf	----	Yolk sac	CM-DiI	No	No
149	28108843	2dpf	32	PVS	Stable transfection	No	Yes
150	28145883	2dpf	----	Yolk sac	CM-DiI	No	No

151	28159748	2dpf	28	PVS	Fast DiI	No	Yes
152	28160553	2dpf	35	Yolk sac	Stable transfection	No	Yes
153	28193911	2dpf	28	PVS	Stable transfection	No	Yes
154	28196873	2dpf	----	PVS	CFSE	No	Yes
155	28209621	2dpf	35	Yolk sac	CM-DiI	No	No
156	28240249	2dpf	----	PVS	Stable transfection	No	No
157	28376864	54hpf	28	PVS	Stable transfection	No	No
158	28420724	2dpf	----	PVS	DiI or Vybrant DiD	No	Yes
159	28465491	2dpf	35	Yolk sac	CM-DiI	No	No
160	28518096	2-3dpf	33	PVS or DoC	Stable transfection	No	No
161	28526577	36hpf	32	Hindbrain-midbrain	Stable transfection	No	No
162	28574600	2dpf	28.5	PVS	DiI	No	No
163	28589491	2dpf	34	PVS	Stable transfection	No	No
164	28606996	2dpf	34	Pericardial	Stable transfection	No	Yes
165	28679777	36hpf	32	Hindbrain-midbrain	Stable transfection	No	No
166	28697174	2dpf	28	PVS	CiI	No	Yes
167	28718729	2dpf	----	PVS	CM-DiI	No	No
168	28790117	2dpf	35	Yolk sac	CM-DiI	No	No
169	28835536	2dpf	34	PVS	DiI	No	No
170	28878163	52hpf	31	Yolk sac & Brain	Stable transfection	No	Yes
171	28892043	2dpf	35	Yolk sac	CM-DiI	No	No
172	28900283	2dpf	----	Yolk sac	CM-DiI	No	No
173	28943451	2dpf	28.5	Yolk sac	CM-DiI	No	No
174	29085081	3dpf	33-34	Intracranial	Stable transfection	No	No
175	29089623	2dpf	35	Yolk sac	CMTMR	No	Yes
176	29141689	2dpf	32	Yolk sac	CM-DiI	No	No
177	29172540	2dpf	35	PVS	CM-DiI	No	No
178	28394345	2dpf	33	DoC	Stable transfection	No	Yes
179	28454577	2dpf	28	DoC	Stable transfection	No	No
180	29106602	36hpf	32	Hindbrain-midbrain	Stable transfection	No	Yes
181	28991224	2dpf	30	PVS	CM-DiI	No	No
182	28925392	2dpf	34	PVS or DoC	DiI	No	No
183	28949016	2dpf	35	Yolk sac	DiI	No	Yes
184	29115578	2dpf	28-32	Yolk sac	QTracker 525	No	No
185	29246646	2dpf	28.5	Yolk sac	Red fluorescence	No	No
186	29291719	2dpf	36	Yolk sac	Stable transfection	No	No
187	29321662	2dpf	36	Yolk sac	Stable transfection	No	Yes
188	29433678	2dpf	----	Yolks sac	CM-DiI	No	Yes
189	29507700	2dpf	----	Yolk sac	CM-DiI	No	Yes
190	29515255	2dpf	34	Yolk sac	CM-DiI	No	No
191	29541384	2dpf	35	Yolk sac	CM-DiI	No	No
192	29545193	2dpf	33	PVS	CM-DiI	No	No
193	29604056	2dpf	33	DoC	Stable transfection	No	No
194	29641204	2dpf	35	Yolk sac	CM-DiI	No	No
195	29681541	2dpf	33	DoC	Stable transfection	No	No
196	29712618	2dpf	33	Pericardial	Stable transfection	Yes	Yes
197	29777274	2dpf	----	PVS	CM-DiI	No	No
198	29849132	2dpf	33	PVS	CM-DiI	No	No
199	29899843	2dpf	33	Yolk sac	CM-DiI	No	No
200	29993186	2dpf	----	Yolk sac	CM-DiI	No	Yes
201	30102771	2dpf	35.5	PVS	Stable trasnfection	No	Yes
202	30195867	2dpf	32	Yolk sac	CM-DiI	No	No
203	30205168	2dpf	28.5	Yolk sac	CM-DiI	No	No
204	30326259	2dpf	35	Yolk sac	CM-DiI	No	No
205	30332851	2dpf	----	PVS	DiI	No	No

206	30339727	2dpf	33	Yolk sac	DiI	No	Yes
207	30367145	2dpf	35	Yolk sac	CM-DiI	No	No
208	30389143	2dpf	----	Hindbrain ventricle	CM-DiI	No	Yes
209	30396905	2dpf	34	PVS	CellTracker green or Hoechst	Yes	Yes
210	30478450	2dpf	35	Yolk sac	CMTPX-Red	No	No
211	30398868	2dpf	----	PVS	DiI	No	No
212	30507376	1dpf	28	Yolk sac	CM-DiI	No	No
213	30484103	2dpf	35	Yolk sac	Stable transfection	No	No
214	30544196	2dpf	32	PVS	CM-DiI	No	No
215	30581541	2dpf	28	PVS	DiI	No	No
216	30473782	2dpf	33	Yolk sac and DoC	DiI	No	No
217	30616104	2dpf	35	Yolk sac	CMFDA dye	No	No
218	30618758	2dpf	28.5	Yolk sac	DiI	No	No
219	30599417	2dpf	----	PVS	DiI	No	No
220	30643816	2dpf	35	PVS	CM-DiI	No	No
221	30657763	2dpf	----	Yolk sac	CM-DiI	No	Yes
222	30684465	2dpf	----	PVS	DiI	No	Yes
223	30720231	2dpf	----	Yolk sac	CM-DiI	Yes	Yes
224	30784915	2dpf	----	SIV	DiI	No	No
225	30787324	2dpf	34	DoC	CM-DiI	No	Yes
226	30968154	2dpf	32	Cardinal vein	Stable transfection	No	Yes
227	30929607	2dpf	28	DoC	Stable transfection	No	No
228	31024847	2dpf	32.5	Pericardial	CFSE	No	Yes
229	31069942	2dpf	33	Yolk sac	CM-RED	No	No
230	31085547	2dpf	33	DoC	CellTrace Far Red	No	No
231	31107449	2dpf	32	Yolk sac	Stable Transfection	No	No
232	31057328	2dpf	35	PVS	Stable transfection	No	No
233	31132644	4hpf	----	Cell mass	Stable transfection	No	No
234	31115172	2dpf	28	Yolk sac	DiI	No	No
235	31138874	2dpf	32	Yolk sac	CM-DiI or CM-DiD	No	Yes
236	31141996	2dpf	28 to 37	Yolk sac	CM-DiI or CFSE	No	Yes
237	31164413	2dpf	33	Yolk sac	DiI	No	No
238	31202990	2dpf	----	Yolk sac	CM-DiI	No	No
239	31213909	2dpf	35	PVS	CM-DiI	No	No
240	31238903	2dpf	33	DoC	Stable transfection	Yes	Yes
241	31221787	2dpf	28.5	Yolk sac	CM-DiI	No	Yes
242	31311575	2dpf	34	Yolk sac	DiI	No	Yes
243	31331338	2dpf	34	PVS	CellTrace Far Red	No	No
244	31346515	2dpf	32	Yolk sac	CM-DiI	No	No
245	31361134	4hpf	34	Cell mass	Stable transfection	No	Yes

'able S1. Collection of parameters used in published literature for drug discovery in zebrafish xenografts models.
