# Supplementary material for: ZeOncoTest: Refining and Automating the Zebrafish Xenograft Model for Drug Discovery in Cancer
Source: Pharmaceuticals (Basel). 2019 Dec 24;13(1):1. doi: 10.3390/ph13010001 (PMC7169390; doi:10.3390/ph13010001)
Supplement: Supplementary file 1 [file pharmaceuticals-13-00001-s001.zip › SupplementaryMaterial_ProofRead/SupplemetaryVideo1.pptx]

## Slide 1
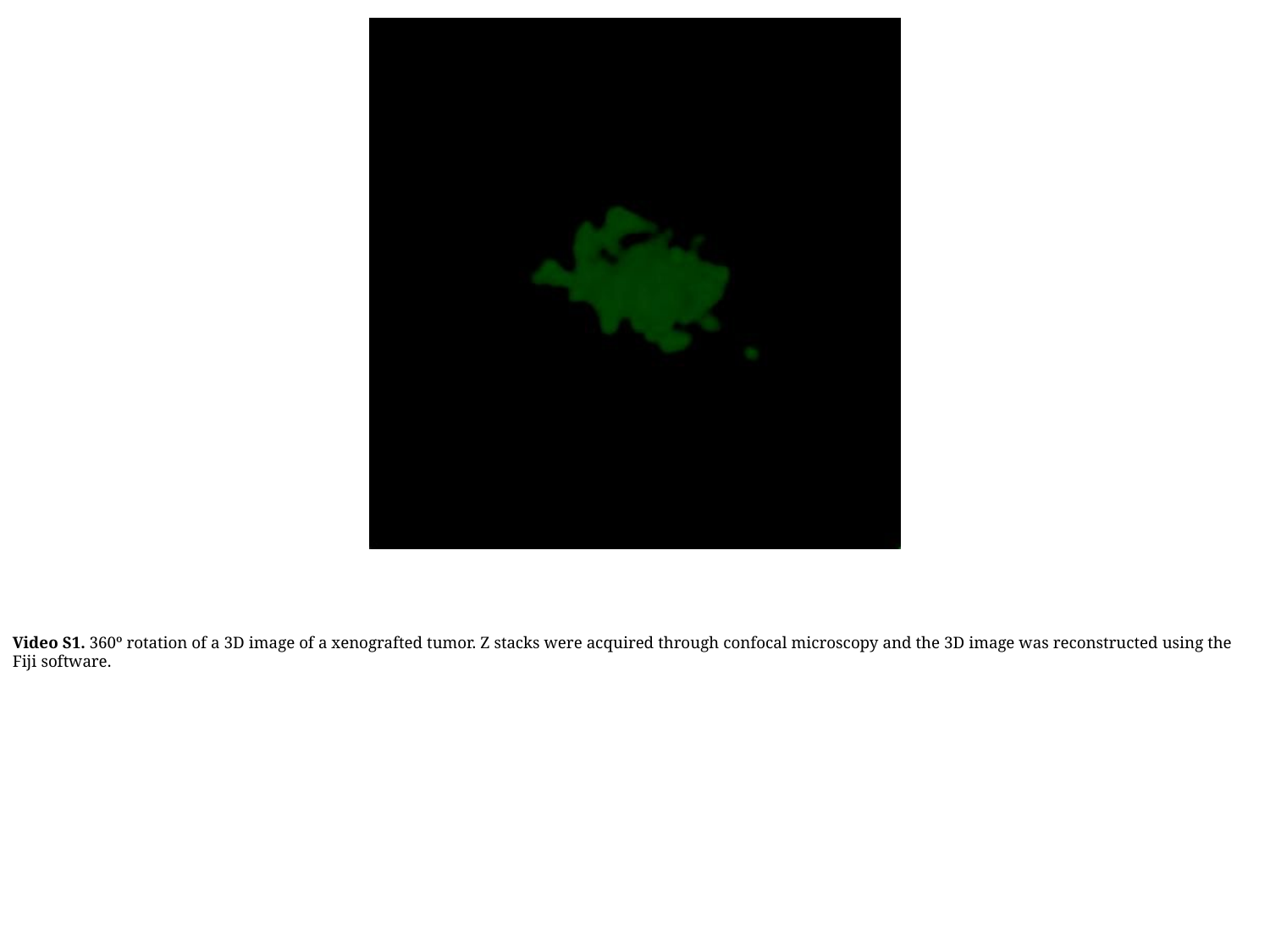

Video S1. 360º rotation of a 3D image of a xenografted tumor. Z stacks were acquired through confocal microscopy and the 3D image was reconstructed using the Fiji software.
